# Haldol Targets IQGAP1 Pathway and Promotes Novel Partner Interactions in Glioblastoma Cell Lines

**DOI:** 10.17912/micropub.biology.000822

**Published:** 2023-05-08

**Authors:** Varun J. Iyer, Mahasin A. Osman

**Affiliations:** 1 Department of Medicine, Division of Oncology, University of Toledo Medical Center, Toledo, Ohio, 43614 United States

## Abstract

Glioblastoma multiform (GBM) is an incurable heterogenous brain cancer with few clinical target options. IQGAP1 is a scaffold oncoprotein involved in GBM with unclear mechanism. Here we report that the antipsychotic drug Haldol differentially alters IQGAP1 signaling and inhibits GBM cell proliferation, thus providing novel molecular signatures for GBM classification and potential targeted therapy in personalized medicine.

**Figure 1. Haldol differentially alters IQGAP1 signaling and inhibits cell proliferation in glioblastoma cell lines.**
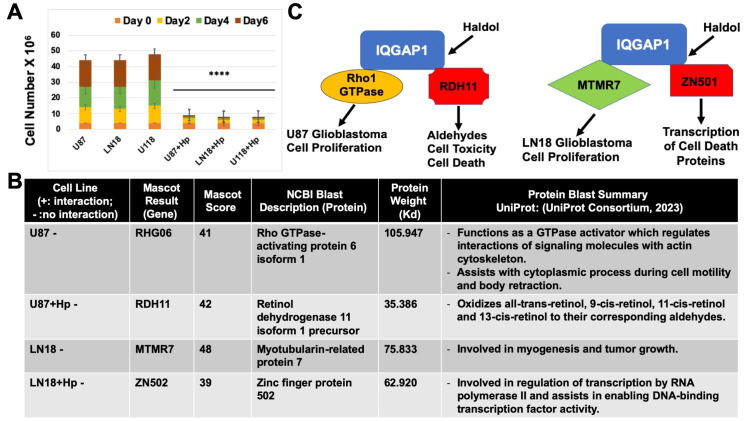
**(A)** Haldol inhibits cell proliferation capacity of the three different glioblastoma cell lines U87, LN18, and U118. Equal number of cells were treated with the drug vehicle DMSO as control or with 10 μM Haldol and the cell density was counted every other day for 6 days. Error bars are the Means ± s.d for *n =* 3, *p-* value **** 0.0001 for the difference between treated and untreated in each cell line **(B)** Protein partner identification by mass spectrometry. Cell lysates from the two glioblastoma cell lines U87 and LN18 that were treated with Haldol or left untreated for control, were immunoprecipitated (IP) with IQGAP1-specific monoclonal antibodies or IgGs as mock control. The resolved IP complexes were processed by MALDI-TOF followed by the Mascot and NCBI Blast software. **(C)** A model of Haldol differential actions on the IQGAP1 signaling pathways leading to inhibition of glioblastoma cell proliferation. The two GBM cell lines, U87 and LN18, are morphologically and molecularly different as suggested by IQGAP1-interacting proteins in Figure B Haldol promoted at least two different sets of interactions per cell line, leading to cell death either via aldehyde toxicity in the U87 cell line, or via transcriptional activation of some cell death proteins involved in apoptosis as in the LN18 cell line. Research is underway to verify these findings mechanistically.

## Description

Glioblastoma multiform (GBM) is an incurable primary brain cancer of heterogenous cellular origin that lacks effective treatment and personalized clinical biomarkers (Ostrom et al., 2014; Haddad et al., 2021) . The overarching goal of this study is to identify clinical biomarkers in GBM. The IQ motif containing GTPase-activating protein 1 (IQGAP1) is an oncoprotein implicated in GBM and other cancers, but its mechanism(s) is incompletely defined. IQGAP1 is a modular signaling scaffold that differentially binds various protein partners, including small GTPases, receptors, kinases, adhesion, cytoskeletal, and motor proteins in a context-dependent manner to nucleate various pathways that control diverse cellular functions like cell dynamics, traffic, and proliferation (White et al., 2012; Osman et al., 2013; Hedman et al., 2015; Osman, 2015) . IQGAP1 localizes to several subcellular organelles, including the plasma membrane, centrosome, nuclear envelope, and the nucleus (Rittmeyer et al., 2008; Osman, 2015; Osman et al., 2020) . Phosphorylation-cycling of IQGAP1 has been shown to be important for its subcellular localization and role in regulating cytokinesis (Wang et al., 2009; Tekletsadik et al., 2012; Osman et al., 2020) . Though IQGAP1 has been heavily studied and a subject of several reviews and book chapters, its emerging role in glioblastoma has been limited to a few studies (Balenci et al., 2006; Rotoli et al., 2019) .

Here, using cell saturation density assays, we identified Haloperidol (Haldol) as an inhibitor of cell proliferation in the three different GBM cell lines U87, LN18, and U118 ( Figure 1A ). Haldol is an FDA-approved drug widely used to treat schizophrenia. To gain a mechanistic understanding of the role of IQGAP1 in glioblastoma and how might Haldol affect IQGAP1 pathways, we performed mass spectrometry on immunoprecipitate (IP) complexes obtained by IQGAP1-specific antibodies from Haldol-treated and untreated U87 and LN18 GBM cell lysates (see Materials and Methods). The results revealed both known and novel protein partners from IQGAP1 IP and not from control IP of the lysates from the two cell lines ( Figure 1B ), supporting the reliability and the specificity of the IP-mass spectrometry analysis. These protein partners differed by cell line as well as by Haldol treatment ( Figure 1B ), thus presenting Haldol as a modulator of IQGAP1 signaling.

In control (vehicle-treated) U87 cell lines, the known IQGAP1 partner Rho GTPase was identified. Rho GTPase is a member of the Ras superfamily of the small GTPases known for its regulation of the cytoskeleton dynamics and cell migration (Nouri et al., 2016) . Binding of the flexible switch region of Rho GTPase to the GAP-related domain (GRD) of IQGAP1 was shown to help activate IQGAP1 (Nouri et al., 2016) . Our results highlight the effect of both proteins on the control or maintenance of cell proliferation in the U87 versus the LN18 cell line, thus presenting a molecular signature that differentiates between these two GBM cell lines. Interestingly, the Myotubularin related protein 7 (MTMR7) was identified as a novel IQGAP1-partner in the untreated LN18 cell line and not in the U87 cell line ( Figure 1B ). The phosphatase MTMR7 has been shown to play a role in myogenesis and tumor growth (Weidner et al., 2020) . MTMR7 forms a complex with the Peroxisome Proliferator-Activated Receptor-gamma (PPARγ) to increase transcriptional activity by inhibiting the extracellular signal-regulated kinase (ERK) binding, which phosphorylates PPARγ (Weidner et al., 2020) . Similarly, IQGAP1 regulates ERK activity through direct binding (Roy et al., 2004; Tekletsadik et al., 2012; Chen et al., 2019) . Thus, MTMR7 and IQGAP1 likely co-regulate cell proliferation by modulating ERK activity and downstream cell proliferation pathways; however, more experiments will be needed to verify this assumption. Additionally, the Myotubularin family is known to control the membrane lipids like phosphatidylinositol-3-phosphatase (PI(3)P) dynamics, to associate with human cancers (Hnia et al., 2012) and to regulate actomyosin activity and cell growth (Hu et al., 2022) . These functions align with the known roles of IQGAP1 in binding membrane phospholipids, interplaying PI3K-Akt and ERK (Chen et al., 2019) , and controlling the actomyosin dynamics to regulate cytokinesis and cell proliferation (Osman, 2015) . Mechanistic studies are underway to understand the dynamic relationship between these newly identified molecules and IQGAP1 in their shared functions.

Treatment with Haldol revealed a different set of IQGAP1 interactions in the two GBM cell lines ( Figure 1B ), suggesting that Haldol modulates IQGAP1-signaling. In Haldol treated U87 cells, the Retinol dehydrogenase 11 isoform 1 precursor (RDH11) was identified. This protein, which localizes to the endoplasmic reticulum, is a member of retinol dehydrogenase enzymes involved in retinoic acid metabolism where it may regulate estrogen and androgen hormones in mammals; however, their function remains highly unexplored (Sahu and Maeda, 2016) and awaits further investigation in relation with IQGAP1. A final product of the RDH11 enzymatic reaction are the reactive species referred to as aldehydes known for their cytotoxicity leading to cell death (Laskar & Younus, 2019), thus presenting a previously unknown involvement for IQGAP1 in aldehyde mediated cell toxicity for further investigation and potential leveraging in GBM treatment.

In the Haldol-treated LN18 GMB cell lines, the Zinc finger protein 502 (ZN502) was identified as an IQGAP1-binding partner ( Figure 1B ). As listed in many databases and gene cards, this understudied protein is located in the nucleus and is predicted to facilitate DNA-binding transcription factors and RNA polymerase II DNA binding activities. This function aligns with the role of IQGAP1 in transcriptional activation via modulating transcriptional co-activators like the Yes-associated protein (YAP) through binding of its IQ domain to the N-terminal domain of YAP (Sayedyahossein et al., 2016) . At the moment, the functional outcome of this novel interaction is unclear and requires further research. However, one can surmise that it activates some apoptotic proteins leading to cell death. Indeed, a recent study concluded that Haldol increases the apoptotic protein caspase-8 in a dose-dependent manner to induce apoptosis (Papadopoulos et al., 2020) but it is unclear if this occurs via IQGAP1 binding. Together, these findings present the possibility of exploiting Haldol-IQGAP1 for molecular classification and targeted treatment of glioblastoma following further studies in preclinical animal models

The results also highlighted the influence of Haldol on cell mass, as gleaned from comparing the protein mass in the untreated and treated cell lines ( Figure 1B ). This finding is consistent with the inhibition of cell proliferation data in Figure 1A and support previous reports that Haldol treatment led to inducing cell cycle arrest and apoptosis in GBM cells via increasing caspase -8-activation (Papadopoulos et al., 2020) . Similarly, Haldol treated neuroblastoma (SK-N-SH) cells exhibited reduction in cell integrity due to the rapid induction of apoptosis via oxidative stress (Gassó et al., 2012). Together these findings support the notion that Haldol alters cell mass (size) leading to cell death via mechanisms involving IQGAP1-dependent transcriptional co-activation.

In summary, using Haldol as a pharmacologic agent, we identified new IQGAP1 partners ( Figure 1C ) potentially useful as diagnostic and clinical biomarkers in GBM biological studies, diagnostics and treatment. Research is underway for more mechanistic studies.

## Methods

**Cell Culture :**

The glioblastoma U87, LN18 and U118 cell lines were purchased from ATCC and grown in DMEM media supplemented with 1% penicillin-streptomycin and 10% fetal bovine serum (FBS) per ATCC instructions. The cells were incubated at 37°C in a sterile humidified incubator supplied with 5% CO _2_ and used at log phase (~ 80% confluency). Cells were discarded after 5 passages and replaced with earlier generations from frozen stocks

**Cell Proliferation Assay**

The classic saturation density assay was used to measure cell proliferation capacity as previously (Wang et al., 2009) . Briefly, glioblastoma cells growing at log phase (80% confluency) were trypsinized and seeded in multiwell plates at 4.5 x 10 ^6^ in triplicatesand incubated overnight to attach. Next morning, the media were replaced by media containing 10 μM Haldol or DMSO vehicle as control. The cells were counted every other day for 6 days, using a hemocytometer or the Countess II (Invitrogen) cell counter.

**Immunoprecipitation (IP) :**

Immune complexes from IQGAP1-specific antibodies vs. IgG controls were analyzed by mass spectrometry to identify IQGAP1-interacting proteins in each condition. Cells growing at ~ 80% confluency were treated with 10μM Haldol or DMSO as a drug vehicle control and incubated in cell culture incubator for 48 hrs. Total cell lysate was prepared by rinsing the cells with ice-cold PBS followed by scraping into ice-cold NP40 lysis buffer [20 mmol/L Tris (pH 8.0), 137 mmol/L NaCl, 1% NP40, 5% glycerol] supplemented with protease inhibitors (1 mmol/L phenylmethylsulfoxide, 10 mg/mL aprotinin, 10 mg/mL leupeptin) and 3 mmol/L Na _3_ VO _4_ . The lysates were cleared by centrifugation and protein concentration was determined by bicinchoninic acid assay (Pierce, Rockford, IL). Four hundred to 1 mL of lysates were pre-cleared with 15 μl of NP40-equilibrated 50% slurry of protein A beads for 1 hr. and used for the immunoprecipitation (IP) reactions. 5 μL primary IQGAP1-specific antibody or IgG as control were added to the cleared cell lysates for IP. The samples were rotated back-to-back for three hours at 4°C followed by the addition of 15 μl agarose bead slurry and the samples were rotated for another three hours and centrifuged at 10,000 rpm for one minute. The supernatant was aspirated and the pellets were washed 3X by adding 1 mL of NP-40 and repeating the centrifugation, and 40 μl of the buffer was added to resuspend the beads. The samples were heated at 100°C for seven minutes and incubated on ice for three minutes before being centrifuged for two minutes and resolved by SDS-PAGE and the gels were Coomassie stained.

**Mass Spectrometry** :

Mass spectrometric peptide analyses was performed using the Coomassie stained in-gel digest with trypsin per Bruker Daltonik instruction manual that was modified from Shevchenko et al, 1996 protocol. Briefly, the gel lanes were carefully cut into 1mm ^2^ pieces via clean scalpel blades and individually placed in Eppendorf tubes and covered with 25 mM NH _4_ HCO _3_ . Samples were vortexed for ten minutes and the supernatant discarded. 25 μl of 10 mM Dithiothreitol was added to each tube, and the samples were vortexed for five minutes followed by supernatant removal, and an hour resting period. Thereafter, 25 μl of 55 mM Iodoacetamide was added, the samples were vortexed, the supernatant removed, and the samples left to rest for 45 minutes. The gels were then dehydrated with 100 μl of 25 mM NH _4_ HCO _3_ in 50% Acetonitrile (ACN), vortexed for five minutes, and rested for 20 minutes. Trypsin (5-25 μl) was added to cover the gel pieces and the samples placed on ice, spun, and incubated overnight. Finally, 1 μl of each sample was loaded onto the MALDI-TOF (Bruker Ultraflextreme) and the corresponding mass to charge (m/z) ratios, spectra, and intensity values were recorded and used for data analyses.

**Data Analyses**

To identify the samples by mass-spectrometry, the MS-Spectrum of each sample was compared to known protein mass-spectra using the Mascot Search Engine. Analyses of the samples were done using the free online Mascot Program within the Protein Pilot Software (Version 5.0.2). Furthermore, UniProt (2022_05 release) and NIH-NCBI Blast Program were used to identify and describe the corresponding proteins identified from each sample.

**Statistical Analyses**

Data are representative of at least three independent experiments with 2-3 replicas each. Statistical analyses were performed using Graph Pad Prism 6.0 (Graph Pad Software, Inc., La Jolla, CA, USA), and the algorithms in Microsoft Excel software (Version 2018) were also used to compare levels between different groups. All statistical tests were two-sided, and *p* values less than 0.05 were considered statistically significant.

## Reagents

**Reagents Information**

**Table:** 

**Cell Lines**	**Origin**	**Description**	**Source**
LN18 (CRL2610)	Cell line isolated from malignant gliomas from a glioblastoma male patient	Adherent cell line and potential to develop tumors	ATCC
U118 (HTB-15)	Cell line isolated from malignant gliomas from a 50-year-old male with a glioblastoma		
U87 (HTB-14)	Cell line isolated from the right temporal lobe of 65-year-old Caucasian male with a glioblastoma		

**Table:** 

**Molecular Tools**	**Function**	**Description/Specification**	**Source**
Acrylamide	Assists in creating gel matrix to separate samples using gel-electrophoresis	Acrylamide Solution (99.9% purity)	Bio-Rad
Ammonium Persulfate	Catalyst for acrylamide polymerization	Oxidizing agent	
Anti-IQGAP1 Primary Antibody	Monoclonal Antibody	Protein derived from C-terminal region of IQGAP1	Thermo-Fisher
Dithiothreitol (DTT)	Disrupt disulfide bonds	C₄H₁₀O₂S₂	
Haloperidol (Haldol)	An antagonist of D2, D3, and D4 dopamine receptor	C _21_ H _23_ ClFNO _2_ Induces apoptosis of neurons in the rat’s striatum	Fisher-Scientific Sigma
HyClone Dulbecco’s Modified Eagle Medium (DMEM)	Basal medium used to sustain cell culture growth	High Glucose medium supplemented with 10% Fetal Bovine Serum (FBS), A/A (antibiotic and antifungal)	
Iodoacetamide (IAA)	Alkylating reagent to assist in protein characterization and mapping	C _2_ H _4_ INO	Thermo-Fisher
Lysis Buffer	Assist in breaking-opening individual cell lines membranes to isolate total proteins	Buffer (20 mL) supplemented with phosphatase inhibitor tablets and 200 μl Protease Inhibitor Solution	
1% NP-40 Cell Lysis Buffer	Serves as lysis and wash buffer to remove impurity from the experimental samples	10 mL NP-40 20 mL IM Tris, pH 8.0 8 g NaCI 100 mL glycerol 0.184 g Sodium vanadate 1L ddH _2_ O	
Precision Plus Protein (SDS-Page Protein Standard)	Used as molecular ruler to identify the weight of each sample	Mixture of 10 known recombinant proteins of varying weights (10-250 kD)	Bio-Rad
Tetramethyl- ethylenediamine (TEMED)	Catalyst for acrylamide polymerization	Used together with ammonium persulfate to prepare acrylamide gel	
Tris Resolving Gel Buffer	Helps to cast the running portion of the SDS-PAGE gel	1.5 M Tris-HCl, pH 8.8	
Tris-buffered saline Tween (TBST)	Wash buffer	Supplemented with 0.1% Tween 20 detergent	Thermo-Fisher
Trypsin	An enzyme used to digest proteins into peptides to facilitate protein analyses prior to running mass spectrometric analysis	Manufactured to resist autolytic digestion Undergoes TPCK treatment to provide specificity. Purified by affinity chromatography prior to shipping.	Promega
